# Supplementary material for: Energy optimization induces predictive-coding properties in a multi-compartment spiking neural network model
Source: PLoS Comput Biol. 2025 Jun 10;21(6):e1013112. doi: 10.1371/journal.pcbi.1013112 (PMC12180623; doi:10.1371/journal.pcbi.1013112)
Supplement: S1 Text — (PDF) [file pcbi.1013112.s001.pdf]

S Supporting Text

S.1 Euler Approximations

The equations (1), (2), (5), and (6) are approximated using standard Euler forward method, with (1) as:

$$V_{a,i}^{l}(t+1) = \alpha_{va} V_{a,i}^{l}(t) + \sum_{j} W_{ij}^{FB} S_{j}^{l+1}(t), \tag{1}$$

where $S_j^{l+1}(t)$ is 1 if the neuron meets the firing condition at time t and 0 else and where $\alpha_{va} = \exp(-1/\tau_{v,a})$. Then, (2) as:

$$V_{s,i}^{l}(t+1) = \alpha_{vs} V_{s,i}^{l}(t) \sum_{j} W_{ij}^{FF} S_{j}^{l-1}(t) + f\left(V_{a,i}^{l}(t)\right) - b_i^l(t) S_i^l(t), \tag{2}$$

where $S_i^l(t)$ is 1 if the neuron i meets the firing condition at time t and 0 else and where $\alpha_{vs} = \exp(-1/\tau_{v,a})$. Equation (5) as:

$$\eta_i^l(t+1) = \alpha_\eta \eta_i^l(t) + S_i^l(t), \tag{3}$$

where $S_i^l(t)$ is 1 if the neuron meets the firing condition at time t and 0 else and where $\alpha_\eta = \exp(-1/\tau_{v,a})$. And Equation (6) as:

$$V_{i,mem}(t+1) = \alpha_{vm} V_{i,mem}(t) + \sum_{j} W_{ij}^{FF} S_{j}^{l-1}(t), \tag{4}$$

and where $\alpha_{vm} = \exp(-1/\tau_{v,mem})$.
