# Supplementary material for: Energy optimization induces predictive-coding properties in a multi-compartment spiking neural network model
Source: PLoS Comput Biol. 2025 Jun 10;21(6):e1013112. doi: 10.1371/journal.pcbi.1013112 (PMC12180623; doi:10.1371/journal.pcbi.1013112)
Supplement: S1 Table — (PDF) [file pcbi.1013112.s002.pdf]

S Supporting Tables

Value	Statistic	p-value
L2 apical tuft	0.334	< 0.001
L2 soma	0.196	< 0.001
L2 δR	0.0246	< 0.001

Table S.1. Kurskal Wallis Test statistics for distributions in Fig.2c, d, e
