# Supplementary figures and images for: Energy optimization induces predictive-coding properties in a multi-compartment spiking neural network model
Source: PLoS Comput Biol. 2025 Jun 10;21(6):e1013112. doi: 10.1371/journal.pcbi.1013112 (PMC12180623; doi:10.1371/journal.pcbi.1013112)

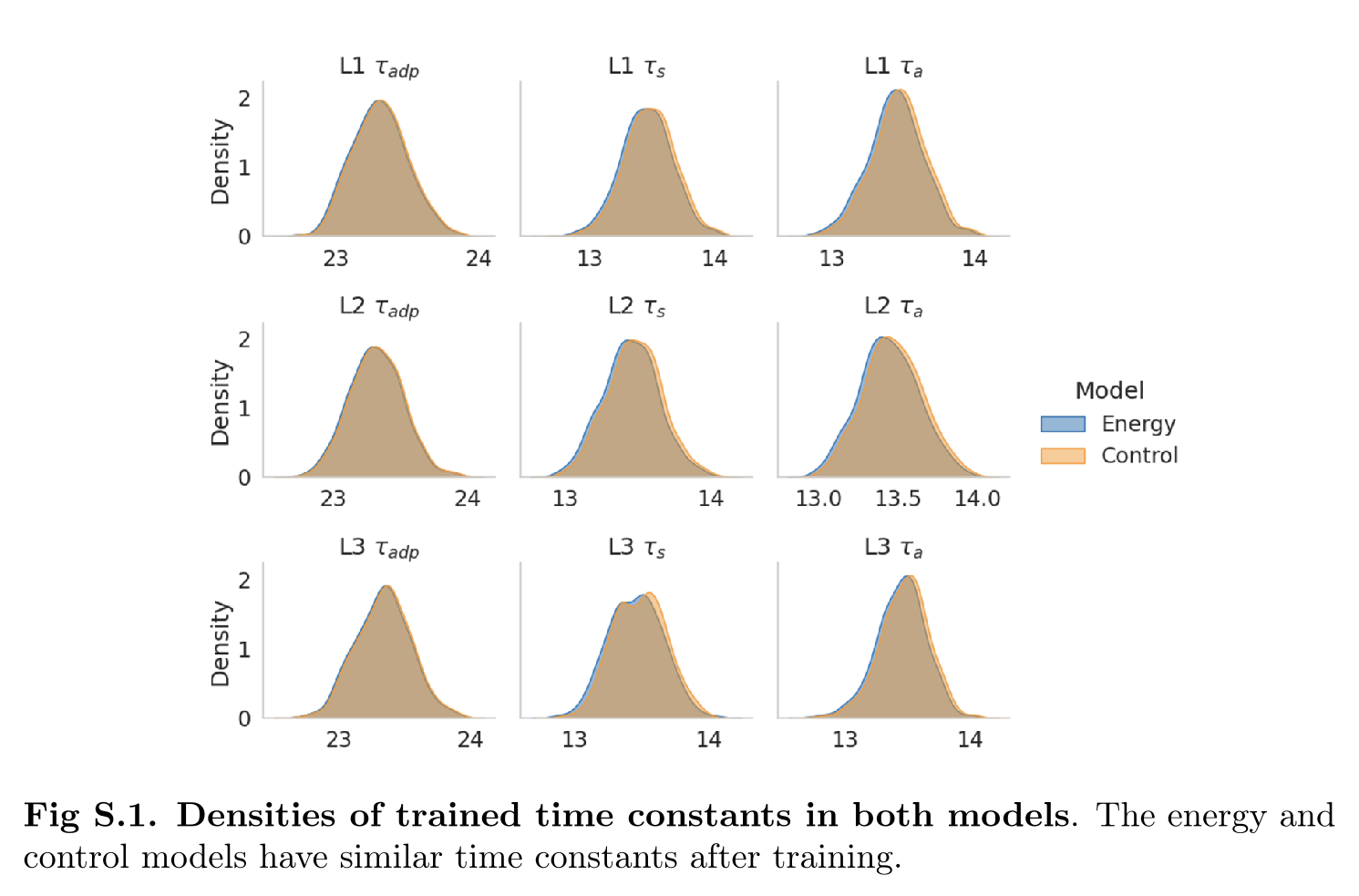

Supplement: S1 Fig — The energy and control models have similar time constants after training. (TIF) [file pcbi.1013112.s003.tiff]

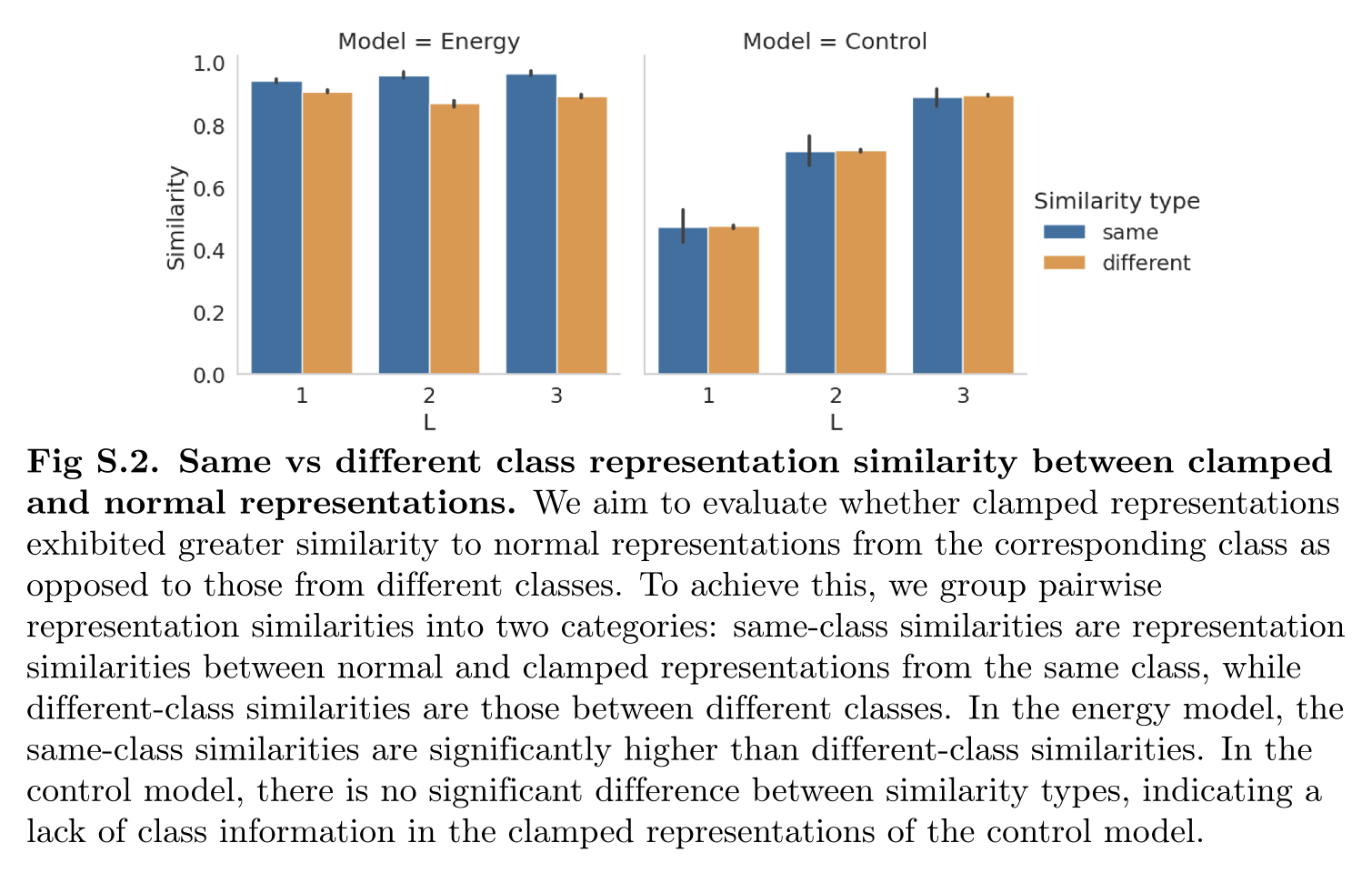

Supplement: S2 Fig — We aim to evaluate whether clamped representations exhibited greater similarity to normal representations from the corresponding class as opposed to those from different classes. To achieve this, we group pairwise representation similarities into two categories: same-class similarities are representation similarities between normal and clamped representations from the same class, while different-class similarities are those between different classes. In the energy model, the same-class similarities are significantly higher than different-class similarities. In the control model, there is no significant difference between similarity types, indicating a lack of class information in the clamped representations of the control model. (TIF) [file pcbi.1013112.s004.tiff]

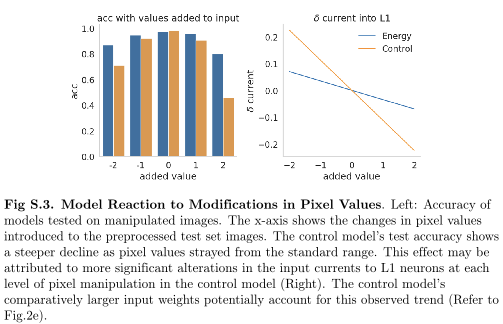

Supplement: S3 Fig — Left: Accuracy of models tested on manipulated images. The x-axis shows the changes in pixel values introduced to the preprocessed test set images. The control model’s test accuracy shows a steeper decline as pixel values strayed from the standard range. This effect may be attributed to more significant alterations in the input currents to L1 neurons at each level of pixel manipulation in the control model (Right). The control model’s comparatively larger input weights potentially account for this observed trend (Refer to Fig 2E). (TIF) [file pcbi.1013112.s005.tiff]

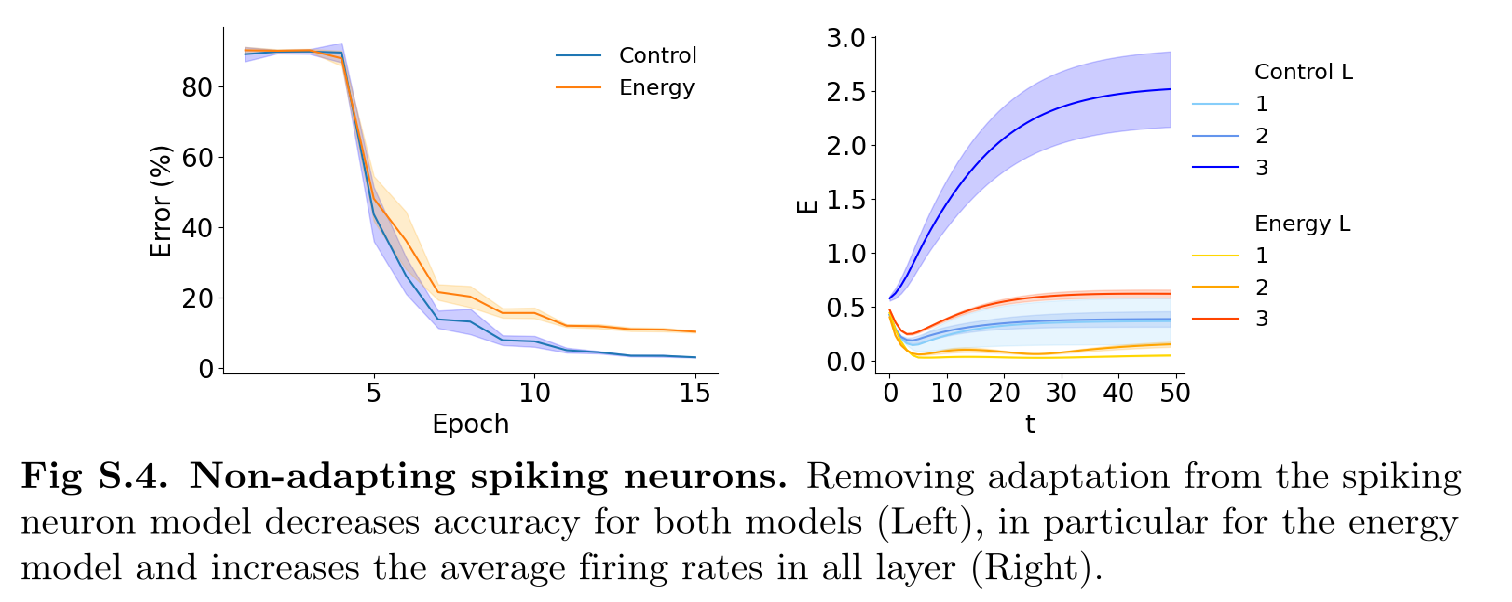

Supplement: S4 Fig — Removing adaptation from the spiking neuron model decreases accuracy for both models (Left), in particular for the energy model and increases the average firing rates in all layer (Right). (TIF) [file pcbi.1013112.s006.tiff]
