# Supplementary material for: Energy optimization induces predictive-coding properties in a multi-compartment spiking neural network model
Source: PLoS Comput Biol. 2025 Jun 10;21(6):e1013112. doi: 10.1371/journal.pcbi.1013112 (PMC12180623; doi:10.1371/journal.pcbi.1013112)
Supplement: Attachment — Submitted filename: Reply_to_Reviewers.pdf [file pcbi.1013112.s007.pdf]

Detailed Replies to Reviewers

Reply to Reviewer 1

Reviewer 1: ***The results are encouraging, but the paper would have greater impact if the results were generalised beyond what is currently presented in the paper, justifying a major revision. A major novelty is the introduction of energy, described in eq (7), which justifies the use of two compartment models. However, some results do not seem surprising (e.g. the caption of Figure 2 reads "The energy model optimises for energy compared to control. ") and "Neurons in the energy model respond differentially to expected vs unexpected stimuli".***

We thank the reviewer for the positive, concise and constructive review, as well as the suggested experiments to further study the model. We carried out these experiments to the best of our ability, and below detail the results and how we included these results in this new version of the manuscript.

Have you checked whether these effects are progressive as a function of α_E, or whether they appear after a certain threshold?

We did indeed check, and we found a gradual trade-off between accuracy and the strength of energy loss (more energy loss, less activity, less accuracy), where we selected our alpha as a trade-off where good accuracy was still achieved. We compared this energy model with a control model that achieved the same accuracy, obtained through slightly earlier stopping.

We remark on this now in the following way:

"We find that this decrease in accuracy for the energy-networks is gradual as a function of the strength of energy-regularization, that is, a larger α setting results in fewer spikes and lower accuracy. This finding is consistent with literature [Boutin et al., 2020] and we selected a value for α that still resulted in high accuracy." (p7).

Moreover, we added a brief discussion on the reason for this in the Discussion:

"Including energy minimization between top-down and bottom-up representations also implies that the network optimizes the somewhat conflicting objectives of learning top-down class-generic representations and bottom-up sample-specific representations. This conflicting objective likely contributes to the accuracy decreasing as a result of the energy loss." (p15)

As in other papers (such as `doi:10.1162/neco_a_01325`), the addition of this code inevitably reduces accuracy (as observed in the lower error rates in the control), so other measures of the benefits of predictive coding should be put forward:
1. Stability to contrast levels or noise is a good place to start,

We investigated this suggestion by adding different levels of Gaussian noise to test samples, for both energy and control models. We unfortunately find that both models degrade in accuracy along identical curves, and there is no advantage to having an energy-model here. Because of this, we did not incorporate this new-but-negative finding in the manuscript.

2. but you could also investigate the effect of feedback on the neural response, for example by looking at whether the presentation of a sequence of digits would differentially encode different inducers of the same digit (e.g. an instance of "3" followed by another of "3") or different digits ("3" - "7"). You can probably guess that the effect would be similar to that observed in mismatch negativity.

We also investigated this suggestion. Specifically, within the setting of Match-Mismatch as shown in Figure 5, we presented one digit, say a "3" for 50 steps, and then presented either another digit, say a "7", or another sample of a "3". For each combination of class sample followed by any other class

sample vs another sample from the same class, we calculated the response difference between energy and control model in the second 50 timesteps.
We unfortunately find that in this specific setting, there was no significant difference between the energy and control models. This suggests that top-down modulation from the output-layer is of relatively short duration, especially as our MSD is measured over 50 timesteps. Again, we did not incorporate this new-but-negative finding in the manuscript.

3. Going further, one could actually test a prediction by titrating the uncertainty of a guess by clamping the output to a single hot output modulated by a contrast, such as (.05, .05, .05, .05, .55, .05, .05, .05, .05, .05, .05, .05, .05) and observing the activity to a novel stimulus.

We also investigated this suggestion, as suggested, by decreasing the clamped output modulation of the selected class, and spreading the decreased activity as an increased input into the other clamped output classes. We find that with increased uncertainty the Mismatch modulation smoothly decreases. We include this result as Figure 5h, noting the approach and results as follows:

"The MSD measure also enables us to predict the effect of a purported increase in classification uncertainty: we decrease the amount of top-down clamping of during stimulus representation, while equally distributed this decrease to increased activation of the other classes. As shown in Fig 5h, the result of this manipulation is a smooth and substantial decrease of the mismatch difference in the energy model, while there is barely a change in the control model." (p12) and, in the caption:
"(h) MSD responses decrease more strongly for the energy model compared to the control model, as a function of decreased top-down clamping, for both somatic and tuft response." (p12).

While not entirely unsurprising, we believe this is a prediction of the model that can be compared in future experiments and we added a remark as such in the Discussion: "where our model additionally predicts that increasing classification uncertainty is associated with decreased differential responses" (p15).

Additional comments:
First, the model is quite complex and uses elements from previous work by the same authors. While FPTT is compared with BPTT, the effect of the parameters of ALIF (especially by turning off adaptation) was not tested and could be a nice addition.

We explored this suggestion, as the ALIF neuron can be very easily turned into a LIF neuron. We find that with this modification, the Energy model has great difficulty achieving decent accuracy (while the Control model also deteriorates). We note this as follows in the text:

"Having adaptive thresholds was critical, as removing adaptation by setting $\beta = 0$ in (4) resulted in poor accuracy and significantly increased firing rates, both for the control model and in particular for the energy model (Fig. S.4)" (p8),

We also added Supplementary Figure S4 that shows this behavior.

2 Secondly, the clarity of Figure 6c could be improved as it is difficult to relate the bars to each other. You seem to want to show that the curves are less saturated - so perhaps you could do fits with saturation functions (e.g. naka-Rushton curves $= 1/(1 + (x/x_50) * * -n))$ ***and plot the histogram of slopes n***

We agree with the reviewer here that the plot as presented is rather qualitative and not too easy to interpret. We changed the plot as follows: For each sample neuron we have 5 data points, corresponding to different amounts of input modulation. For each neuron with these 5 data points, we calculated a linear fit and r2 between modulation and change in firing rate. We find the linear fit to be excellent, with all r2's >0.98 (and most >0.99). We then plotted a sorted bar chart of this slope for all 20 sample neurons, showing that most neurons have a negative modulation of similar quantity, with

some outliers. We modified the manuscript as follows: we changed the caption text for figure 6c to read:

"(c) Slope for the spike rate as a function of input modulation (dS/dA) for 20 individually sampled neurons in L1 of the energy model. Most sampled neurons show a graded response in spike rate to input pixel manipulation." (p13).

We comment on this in the Result 3.4 as follows:

"in Fig.6c, we plot for 20 randomly selected neurons in L1 the slope for the change in firing rate as a function of modulation of the input. We applied 5 different levels of input modulation and measured the change in firing rate for each level. We used linear regression to fit this change to a linear response model, obtaining excellent fits with $r2 > 0.98$ for all neurons; Fig.6c plots the slope for each of the 20 neurons." (p13)

3 Also, what would be the guessed numbers in Fig. 4 from these reconstructions?

We added the guessed number into the figure for each sample, and note this in the caption.

4 Fig. S.4. should be made more quantitative by plotting some sort of "tuning curve" of this neuron to different bars and see if the control shows the same behaviour.

The reviewer is right in that the figure is rather qualitative and also does not directly contribute to the points we try to make in the manuscript; we also notice that because of this we already removed all links to this figure in the main text, but accidentally kept the figure, apologies for this. We now removed this SI figure.

5 Finally, I would have many questions regarding using other datasets than a static one (MNIST), yet it seems perhaps going beyong the scope of this paper.

While we expound on this topic implicitly in the Discussion when comparing to the work of Ali et al, we would indeed argue that this goes beyond the scope of our study, as we argue that the problem of dynamic datasets processed in continuous time, at least in the setting of stimulus switches, essentially becomes a decision-making problem with all added complications. To elaborate on this point, we added the following sentence: "Combining learning with decision-making could however be a way to study dynamic dataset in future work." (p16)

Minor comments

1 p1 Check and simplify the syntax of this long sentence "Here, we demonstrate how recurrent networks comprised of multi-compartment ..."

Good point. We rephrased this as follows: "Defining an internal energy-measure for multi-compartment spiking neurons, we demonstrate how the resulting recurrent networks can exhibit several predictive coding like-properties when optimizing for both task and energy efficiency." p1.

2 p2 "that when when optimizing" > "that when optimizing"

Thank you, corrected

3 p5 Eq (3) is equivalent to $1/4 \tanh(2x)$ which is commonly used in other models. or maybe simply tanh as weights would scale activity ?

The reviewer is correct in that our equation is more easily recognized as the scaled tanh function ($1/4 \tanh(x/2)$), and we modified the equation accordingly.

4. Fig 2. (d) misses labels on both axis. complete "figures plot 95%ci." > "figures plot 95% confidence intervals."

Thank you, corrected.

5. Fig3 could be improved by showing bigger images + it could be useful to show the guess given by the inference for these generated images

We rearranged the figures to increase vertical space and reduce white-space, which increased the figures. We also included the top-3 classification guess for each decoded representation, explained in the caption of Fig 3 as: "Below each decoded digit, the top 3 classification as obtained from the respective models given these digits as input." (p9)

6. Fig 6: the axis labels are not written in proper English, and the legend should be made more explicit (reading the paper makes it obvious what E vs nE are but readers may get confused "Energy-based" and "Control" could be more informative). y-labels in (c) are not necessary "(b) Spike rates of 20 individually sampled neurons in L1 of the energy" − > "(c) Spike rates of 20 individually sampled neurons in L1 of the energy"

Thank you, we corrected that in the new version of the figure.

Reply to reviewer 2

R2: While the manuscript is overall easy to understand there are some minor issues with the description of the methods that need to be fixed before publication and some minor writing issues.

We thank the reviewer for the kind words and positive review. Below we outline how we corrected the noted minor issues.

Methods:

1. The description of the neural network model is inconsistent and mathematically incorrect. The authors define $S_i(t)$ as taking values 0 or 1, the latter when a neuron's voltage reaches the firing threshold, at which time the neuron is instantaneously reset to 0 by subtracting the threshold. Subsequently $S_i(t)$ is used on the right hand side of ODEs for the voltages. However, with this definition, they have no effect on the voltage (the set of times $S_i(t)$ is non-zero is a null set and finite value 1 hence has no influence). I believe the authors are mixing a continuous time description in their methods section with the discrete time implementation in their code to arrive at this inconsistent/incorrect description.

The reviewer is absolutely correct! Thank you for noticing, apologies, at some point we seemingly start to mix code approximations and the underlying dynamical system. We reformulated the equations more classically as continuous dynamical systems subject to Direct-delta spikes, and added a section in the Appendix S1 where we write down the Euler forward approximation of these continuous functions and noted in the Methods section that we used the Euler forward method (p5).

2. On page 5, fully connected weights (with bias): What does "with bias" mean? Please define unambiguously by a formula.

We added the detail: "where a bias is implemented for each neuron as a constant current injection through a trainable weight, $W_{ib} \cdot 1$, ie as in [Yin et al., 2023]" (p5)

3. "After spiking, the somatic potential undergoes a soft reset of the current value of $b_i^l(t)$ (Eq. 2), retaining the amount in the potential that exceeds the threshold due to the

time step effect." (p5) $->$ everything is described in continuous time, so this statement makes no sense (see 1 above, same issue of mixed descriptions).

Thank you, we corrected this description.

4. The Methods section does not elaborate how the presented ODEs are solved numerically.

As noted above, we added the sentence "The equations (1), (2), (5), and (6) are calculated using the Euler forward method, see Appendix S.1." (p5).

5. It would be helpful to already mention explicitly in the methods that timescales (τ_x) are individual to neurons and also subject to training

This was indeed omitted. For clarity, we added: "Training adjusts all weights W as well as all decay-constants τ, the latter specific for each individual neuron." (p5)

6. On page 7, formatting of "5e-2" should be in maths notation

Thank you, fixed.

7. "At the beginning of inference for each batch of samples, spiking neurons are initialised with somatic membrane potentials uniformly distributed between 0 and 1 at the beginning of training." $->$ broken sentence

Thank you, corrected.

8. "all bias terms were initialised to 0 prior to training." $->$ as above, please explain explicitly how biases work in your SNN model

We added the specification $W_{ib} = 0$, to link to the added earlier explanation (p5).

In the results:

1. The classification error rates achieved appear to differ in a statistically significant way - please add commentary on this in the paper.

This was also noted by reviewer 1, and we now argue, also based on the observation of the effect of changing the relative strength of generative vs discriminative losses, that we are optimizing for conflicting goals. We comment on this as follows in the discussion:

"Including energy minimization between top-down and bottom-up representations also implies that the network optimizes the somewhat conflicting objectives of learning top-down class-generic representations and bottom-up sample-specific representations. This conflicting objective likely contributes to the accuracy decreasing as a result of the energy loss." (p15)

2. Fig 5 caption: "on either end of stimulus onset.": The stimulus onset is the point in time when the stimulus starts; I believe the authors meant "stimulus presentation". This confusion repeats several times throughout the paper

The reviewer is correct, apologies for the oversight. We amended the text and corrected Fig 5b and 5g.

3. Please define the Mean Signed Difference by an explicit equation.

We added the definition and equation (9). (p11)

4. “response also varies less than the control model in for the same amount”– > broken sentence

Indeed, corrected to: “The energy model’s response also varies less than the control model for the same amount”

References

[Boutin et al., 2020] Boutin, V., Franciosini, A., Ruffier, F., and Perrinet, L. (2020). Effect of top-down connections in hierarchical sparse coding. *Neural Computation*, 32(11):2279–2309.

[Yin et al., 2023] Yin, B., Corradi, F., and Bohte, S. M. (2023). Accurate online training of dynamical spiking neural networks through forward propagation through time. *Nature Machine Intelligence*.
