# Supplementary material for: Energy optimization induces predictive-coding properties in a multi-compartment spiking neural network model
Source: PLoS Comput Biol. 2025 Jun 10;21(6):e1013112. doi: 10.1371/journal.pcbi.1013112 (PMC12180623; doi:10.1371/journal.pcbi.1013112)
Supplement: Attachment — Submitted filename: Reply_to_Reviewers_PlosCB_Energy_R2.pdf [file pcbi.1013112.s008.pdf]

Detailed Replies to Reviewers

We thank both reviewers for their positive comments, where Reviewer 2 notes his comments and questions have been satisfied, and Reviewer 1 poses some minor additional queries.

Reply to Reviewer 1

Reviewer 1: ***The current analysis provides a foundation for understanding the learning dynamics of the network, but could benefit from additional insights into the structural properties of the network after learning. An examination of weight distributions and connectivity patterns could reveal nuanced computational mechanisms. In particular, an examination of the relative strengths of feedforward and feedback connections may reveal interesting parallels with existing neurophysiological research on neural information processing, such as Bruno and Sackman's research on thalamo-cortical connections.***

From this suggestion, we first carefully inspected the weight matrices of trained models. We find some interesting properties of the networks, however, we do not find significant differences on these measures between energy and control models.
Notably, we first inspected the plots of the trained forward and corresponding backward weight-matrices. Here, after training, we find a subset of neurons where strong forward excitation from one neuron broadly to most targeted neurons is balanced by strong backward inhibition from the targeted neurons to the forward projecting neurons. This observation is similar to that in most deep networks in AI that a limited set of neurons does the heavy lifting to solve the problem.
This links to the related comment by the reviewer:

Reviewer 1: ***Last, the authors could consider exploring the spatial distribution of weights after learning, examining whether feedforward inputs exhibit the anatomically suggested precision and clustering (patchy characteristics), while feedback connections remain more diffuse.***

This is an intriguing suggestion, and we investigated the forward and backward weight matrices in trained models for both energy and control models. Unfortunately we found no significant difference between forward and backward connectivity diffusion, and no difference in this respect between forward and backward projections.
Specifically, since successive neural layers are fully connected, we examined several measures of projection diffusion, the Effective Rank of the weight matrix, the Shannon entropy and the Gini-coefficient. Where for the latter two, we calculate the average value for the connections from a single neuron to the next layer, averaged over all neurons. For all three measures, we find very similar values for the projections as calculated for the energy model and the control model. We also do not find significant differences between forward and backward connections on these measures. Visual inspection of the weight matrix also shows no "patchy" connectivity patterns, but instead, as noted above, shows that subset of neurons is strongly forward connected to all projecting neurons and this same neuron in return receives strong inhibitory projections, to balance out.
This negative finding with regards to connectivity diffusion may be due to the fully connected nature of the connectivity patterns, which provides a highly dispersed starting point for learning. It seems logical to extend the model to more realistic starting points for the connectivity, like a convolutional version of the network.

Taken together, we believe our findings on these investigations suggest that the neural architecture needs to be taken into account in future studies, and we added a note in the Discussion of the manuscript as follows:

"To study spatial organization for instance in thalamocortical circuits (Bruno & Sackmann, 2006), and compare to known biological observations, the fully connected architecture could be replaced by more natural connectivity patterns mimicking receptive fields." (p17)

Reviewer 1: ***Second, the potential role of temporal precision and network performance provides a rich area for further investigation, for example by investigating the role of temporal jitter in the input.***

We investigated how jittering the input and/or all neurons in the network during testing/inference affected the performance of the respective models. We investigated this by creating a stochastic threshold in each spiking neuron, and increasing the stochasticity as a proxy for temporal jitter. We find here a consistent relationship between sensitivity to jitter and average firing rate: the lower the average firing rate in the networks, the more sensitive the networks became to temporal jitter. This held for both energy and control models, where the reported control model was less sensitive compared to the energy model. However, once we regularized the activity in the control model to the same level as that in the energy model, by imposing an L1 loss on the number of spikes, we obtain identical behavior (we can regularize both energy and control models to each use about half the spikes compared to the reported models without loss of accuracy, which for the energy model however came at expense of the generative properties). From the perspective of the study, we thus see no difference between energy and control models with respect to jitter-sensitivity when using essentially equal spikes for processing. The issue is interesting, because one can imagine a situation where top-down predictive spikes precisely cancel bottom-up stimulus-driven spikes. Here again the fully connected architecture imposes that each neuron receives spikes from many other neurons, while more targeted connectivity patterns could support such spike-time-based predictive coding. Also, and here we are speculating, we also feel that a likely reason for this lack of differentiation in terms of temporal sensitivity stems from the inherent "rate coding" that the surrogate gradient learning paradigm imposes. This topic has our attention, and we added a considertion for this in the Discussion:

"Similarly, the surrogate gradient used in the backward pass is principally sensitive to spike-rates; learning rules that are more sensitive to spike-times could be investigated to study the potential for spike-time-based top-down prediction canceling predictable bottom-up inputs (Kim et al, 2020)."

Reviewer 1: ***minor points: Regarding the technical presentation, two minor points require attention. First, the tables (Tables 1 and 2) should include explicit units for all measurements to ensure clarity and reproducibility. Second, the term "inference" appears to be an inappropriate neologism and should be replaced with standard scientific terminology such as "inference" or "inferring".***

We added the units for clarity in Tables 1 and 2 as suggested. With regards to the notion of "inference", we are slightly confused what distinction the reviewer had in mind. To avoid any misunderstanding, we decided to clarify our nomenclature as early as possible in the manuscript: "Adopting the idiom of the deep learning field, we refer to the calculation of neural activity patterns in the network given inputs as "inference"." (p4).
